# Who measures the measurers: Closing the administrative accountability gap in non-profit acute care hospitals

**DOI:** 10.1186/s12913-026-15079-9

**Published:** 2026-07-09

**Authors:** Mahua Dey

**Affiliations:** 1 University of Wisconsin School of Medicine and Public Health, 600 Highland Ave, Madison, WI 53792 USA; 2 Harvard TH Chen School of Public Health, Harvard University Boston, Boston, MA USA

**Keywords:** Hospital administration, Administrative costs, Non-profit hospitals, Principal-agent theory, Health care governance, Management accounting

## Abstract

**Background:**

Administrative costs in U.S. non-profit acute care hospitals are widely reported to be high and rising, yet hospital administrators are largely exempt from the productivity measurement. We introduce the Administrative Accountability Gap (AAG), the systematic exemption of administrative functions from the cost-per-output accountability criteria imposed on every other clinical workforce category and ask whether high administrative cost is structurally inevitable or a governance issue.

**Methods:**

We develop the AAG construct through principal-agent theory and operationalize it using the formally designated Administrative and General (A&G) cost center from CMS HCRIS Worksheet A. We analyzed 6,354 non-profit hospital-year observations across fiscal years 2021–2023. We complement the empirical analysis with an illustrative management accounting framework applied to a model hospital, with sensitivity analysis.

**Results:**

Among 2,142 non-profit acute care hospitals in FY2023, median A&G cost was 17.9% of total cost (Ranged 12.4% (10th percentile) − 25.1% (90th percentile)). Non-profit A&G share (17.9%) differed significantly from for-profit (20.3%) and government (15.4%) hospitals (*p* < 0.001). Using an illustrative model, we show that $0.56 M–$3.64 M in possible avoidable annual costs attributable to a single administrative domain.

**Conclusions:**

Administrative cost intensity varies substantially among otherwise comparable non-profit hospitals, supporting the view that it reflects governance rather than necessity. Existing management accounting tools can close the AAG; we propose a framework and a research agenda for institutional validation. The principal barrier is the structural conflict of interest that allows administrators to design the measurement systems they are exempt from.

**Supplementary Information:**

The online version contains supplementary material available at https://doi.org/10.1186/s12913-026-15079-9.

## Introduction

Hospital cost accountability is profoundly asymmetric. Every morning in U.S. hospitals, nurse managers are called to account for staffing ratios, overtime hours, and cost per patient day. Physicians document every procedure and billable minute. The administrative leaders who design and impose those measurement systems are, in most institutions, subject to no equivalent productivity measurement of their own. We term this asymmetry the Administrative Accountability Gap (AAG): the systematic exemption of hospital administrative functions from the cost-per-output accountability routinely applied to every other frontline clinical workforce category.

The magnitude of administrative cost in U.S. hospitals is contested in the literature, in part because different studies use different definitions. Estimates of hospital administrative cost as a share of expenditure range widely, from roughly 25% to as high as 49% depending on whether the measure captures only formally designated administrative departments or also includes billing, insurance-related administration, and administrative activities embedded within clinical units [1–3]. To obtain a realistic, replicable baseline rather than relying on this dispersed range, we calculated our own estimate directly from US federal cost-report data using the formally designated Administrative and General (A&G) cost center. What is consistent across the literature is the overall direction of the change where over the last two-decade administrative expenditure has grown significantly faster than direct clinical spending, and U.S. hospitals carry administrative burdens substantially higher than comparable institutions in Canada, the United Kingdom, and other U.S. non-profit sectors [1, 4, 5].

Prior literature has documented administrative cost growth, [1, 2, 6] examined principal-agent relationships in health governance, [7, 8] and applied process-improvement methods in clinical settings [9, 10]. However, no existing framework integrates governance theory, management accounting methodology, and empirical cost-structure analysis into a unified, testable account of administrative accountability specific to non-profit acute care hospitals. This paper makes three contributions. First, it operationalizes the AAG as a measurable construct and tests it empirically using national CMS cost-report data. Second, it develops the governance diagnosis through principal-agent theory and connects it directly to the empirical findings. Third, it proposes a symmetric management accounting framework and a research agenda for institutional validation. We focus on non-profit acute care hospitals because their charitable tax exemption creates a fiduciary obligation to direct resources toward patient care, making the AAG a matter of public trust.

## The scale and trajectory of administrative cost

**Fig. 1:**
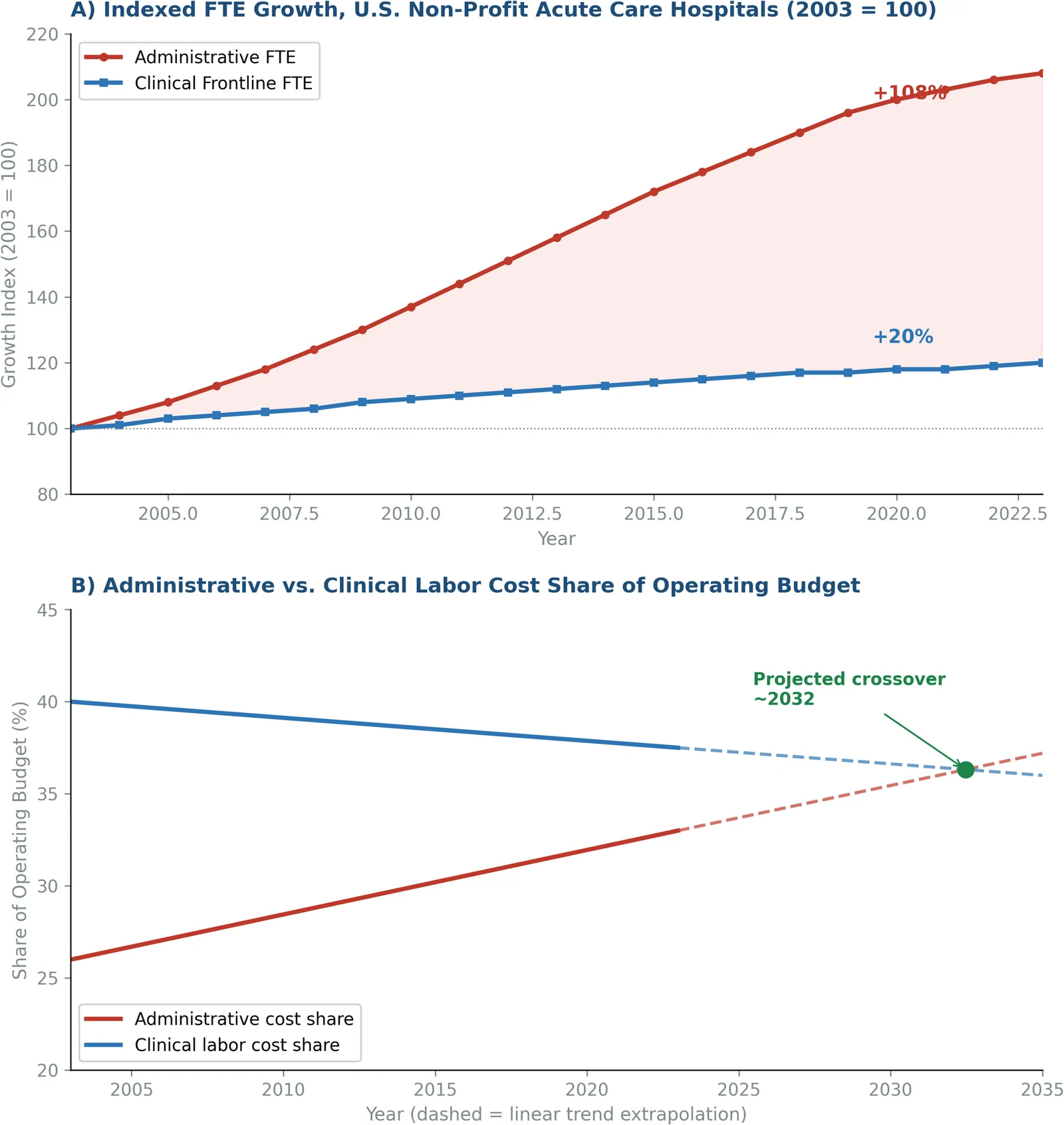
Administrative FTE growth and cost-share trends in U.S. non-profit acute care hospitals. (**A**) Indexed FTE growth (2003 = 100). (**B**) Administrative and clinical labor cost as share of operating budget, with linear trend extrapolation beyond 2023 (dashed). Sources: Woolhandler & Himmelstein (1991); Himmelstein et al. (2020); Handlon & Simpson (2025). *Extrapolation carries uncertainty and assumes continuation of recent linear trends

**Fig. 2:**
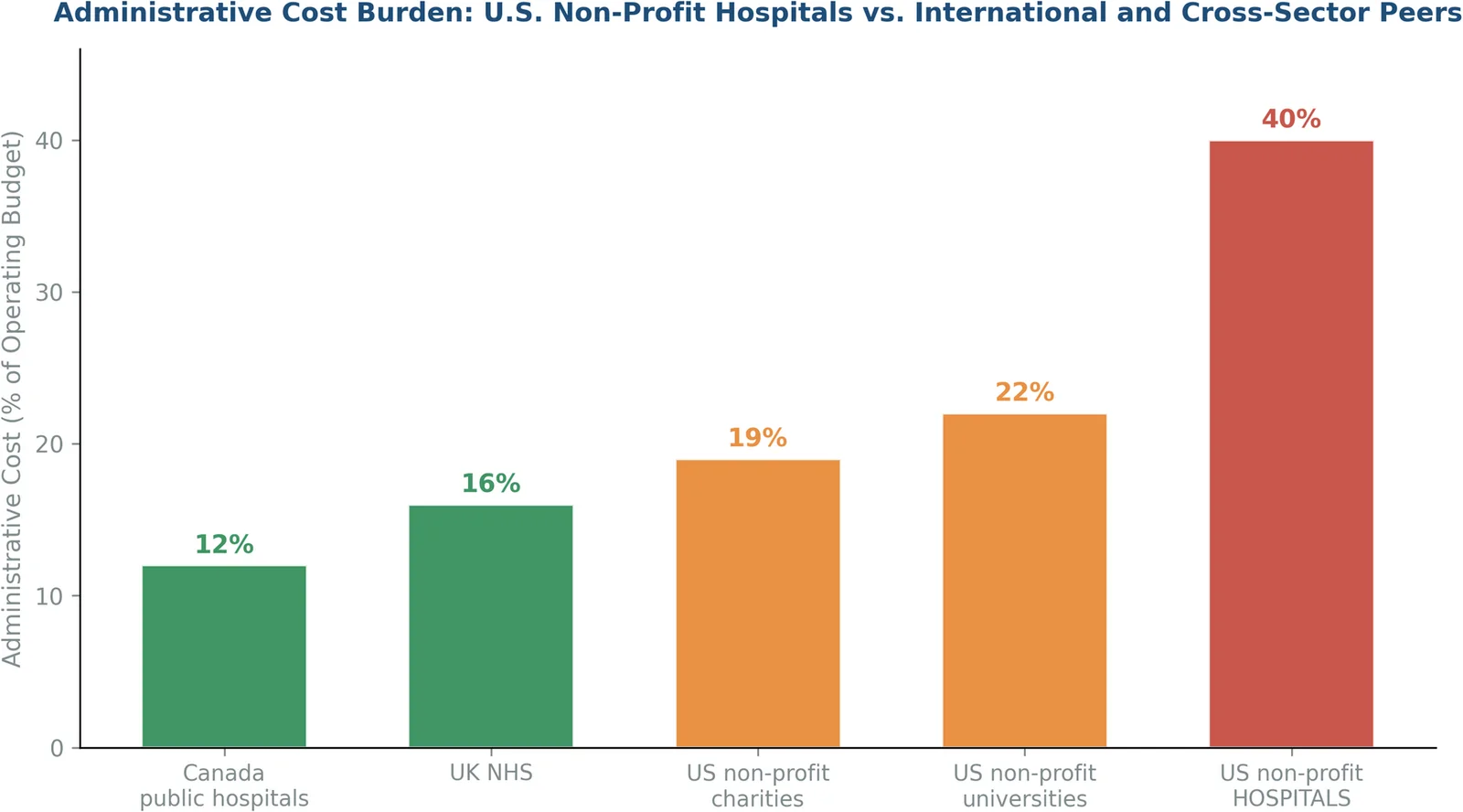
Comparative administrative cost burden: U.S. non-profit hospitals versus international and cross-sector peers. *Author’s figure from Himmelstein et al. (2020); CIHI (2022); NHS England (2022); NCCS (2022); NACUBO (2022)*

Administrative full-time-equivalent (FTE) staff in U.S. non-profit acute care hospitals have grown at more than five times the rate of clinical frontline FTE since 2003, with a corresponding shift in cost share (Fig. 1) [6]. The comparative context is essential to interpretation: U.S. non-profit hospitals carry administrative burdens roughly two to three times those of Canadian public hospitals (~ 12% of operating budget) and well above U.S. non-profit universities (~ 22%) and charitable organizations (~ 19%) (Fig. 2) [1, 4, 11, 12]. The claim that 40%-plus administrative cost is an inevitable feature of acute care operation is not supported by cross-sector comparison a proposition we test directly with hospital-level data below.

## Governance diagnosis: a principal-agent account

### Agency theory as the analytical lens

**Fig. 3:**
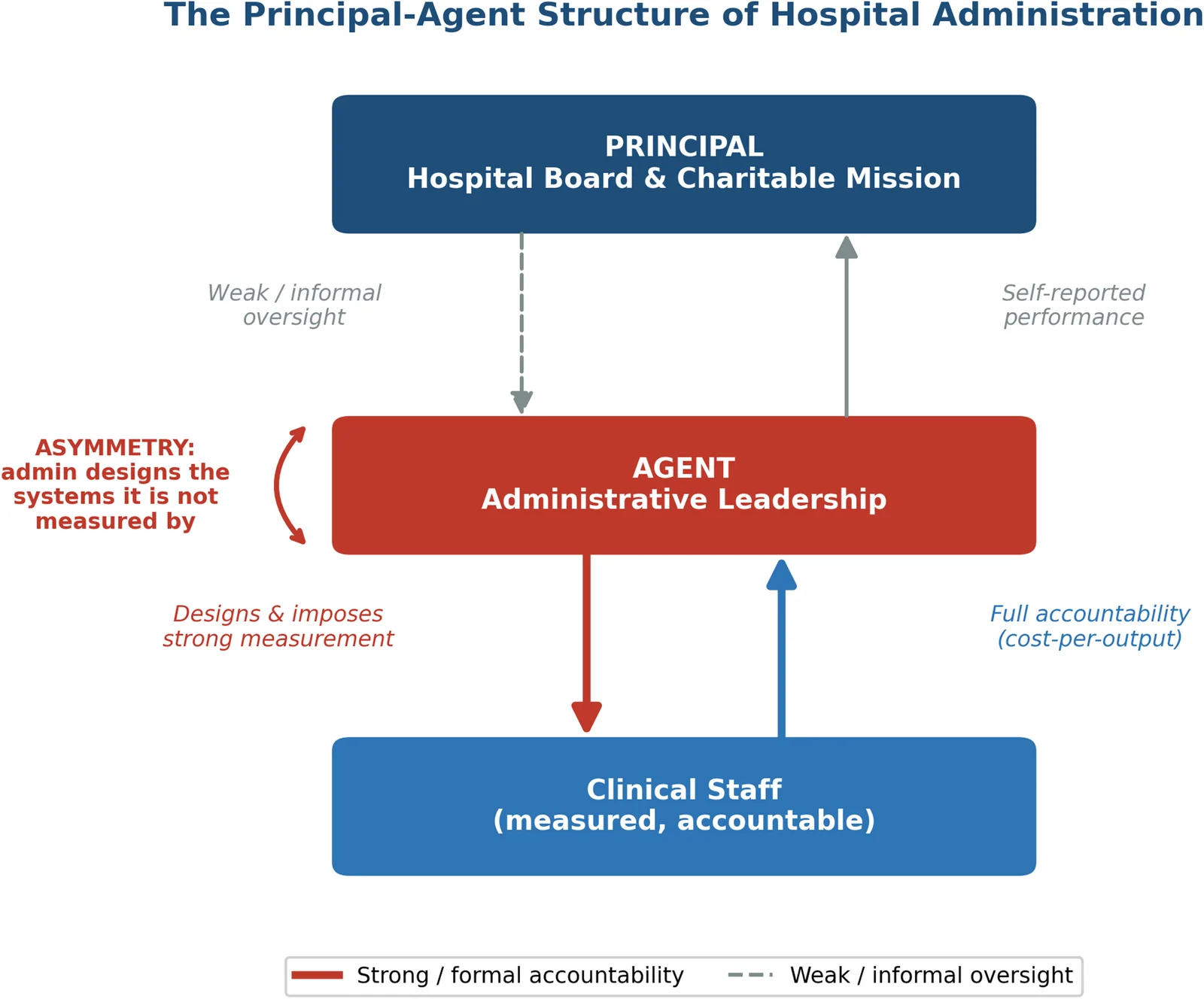
The principal-agent structure of hospital administration. Arrow weight reflects accountability strength: bold solid = formal/strong; dashed = informal/weak. The central asymmetry (red arc) is that administrators design the measurement systems they are not themselves measured by. *Author’s figure*,* adapted from Ross (1973) and Jensen & Meckling (1976)*

Principal-agent theory, developed independently by Ross and Mitnick in 1973 and extended to corporate governance by Jensen and Meckling, describes the conflict that arises when an agent’s incentives diverge from a principal’s and the principal cannot fully observe the agent’s actions [13, 14]. Brinkerhoff and Bossert have demonstrated its applicability to health-system accountability, identifying information asymmetry as the central governance failure mechanism [8]. In the hospital setting, the board and charitable mission are the principal and administrative leadership is the agent. The theory predicts that rational agents will design monitoring systems that minimize scrutiny of their own activities while preserving the appearance of oversight. Figure 3 depicts the resulting structure: administrative leadership simultaneously acts as the board’s agent and as the architect of the performance-measurement systems applied to clinical staff, an unmanaged conflict of interest.

Principal-agent theory is not the only relevant lens to evaluate this scenario. Stewardship theory that views executives as responsible caretaker of an organization’s resources would predict that administrators, as stewards of the mission, will voluntarily pursue transparent accountability. [15]. However, the empirical record of sustained administrative cost growth over decades is more consistent with agency-theory predictions than with stewardship, suggesting that governance structures assuming stewardship behavior may be misaligned for this context. Two complementary frameworks further inform the analysis: (i) sociotechnical systems theory, which holds that accountability outcomes depend jointly on technical measurement systems and the social/organizational context in which they operate [16] and (ii) organizational change theory, which emphasizes that introducing measurement where none existed triggers predictable institutional resistance [17]. These frameworks caution against treating the AAG as a purely technical problem solvable by measurement tools alone. Institutional culture, leadership incentives, and organizational dynamics are co-determinants of accountability that need to be also aligned for implementation.

### AAG arise from four mutually reinforcing mechanisms

1. **Institutional power and information asymmetry**: Administrative leaders control both the design of accountability systems and the committees that approve them, while clinical managers who bear the downstream costs lack authority to challenge administrative cost structures [18].
2. **Professional norm entrenchment**: Treating administrative cost as fixed, unattributable overhead has been codified in hospital accounting practice since the 1976 AHA Chart of Accounts and the Medicare Cost Report framework, generating path dependence that persists beyond its original rationale [19, 20].
3. **Complexity invoked as a measurement barrier**: Administrative departments commonly argue that the strategic and relational nature of their work resists output measurement. Whatever the merits of that argument, its asymmetric acceptance, applied to administrative but not clinical departments managing comparable resources is in itself a governance phenomenon the framework seeks to surface rather than adjudicate [21].
4. **Board capacity limits**: A scoping review of hospital governance accountability structures found that board-level performance monitoring of administrative functions is absent or underdeveloped in the majority of reviewed governance frameworks, limiting the principal’s ability to exercise oversight [22].

These mechanisms are mutually reinforcing where administrative influence shapes what is measured; unmeasured performance cannot be challenged; and boards without analytical infrastructure provide no external check. The framework predicts a specific, testable empirical signature of wide and persistent variation in administrative cost among comparable institutions, reflecting drift in the absence of binding accountability.

## Empirical analysis: administrative cost structure in U.S. non-profit hospitals

### Data and methods

We analyzed the CMS Hospital 2010 Cost Report (HCRIS) for fiscal years 2021–2023. We restricted the sample to short-term acute care hospitals and classified ownership using the HCRIS Type of Control field (Worksheet S-2): codes 1–2 as non-profit, 3–6 as for-profit, and 7–13 as government. Administrative cost was measured as the formally designated A&G cost center (Worksheet A, line 5), expressed as a share of total facility cost (Worksheet A, line for total costs, net column). This is a deliberately conservative, replicable measure: it captures the cost center hospitals themselves designate as administrative and excludes the broader billing and insurance-related administrative activity that produces the higher end of the literature range. After excluding reports with non-positive totals and restricting the share measure to the economically valid 5–40% range to remove cost-allocation artifacts (affecting under 1% of observations), the analytic sample comprised of 6,354 non-profit hospital-year observations (2,142 in FY2023). We report medians and interquartile statistics given right-skewed distributions and test group differences with the Mann-Whitney U test. Analyses was done using Python (Pandas, SciPy).

### Result 1: Administrative cost varies twofold among comparable non-profit hospitals

**Fig. 4:**
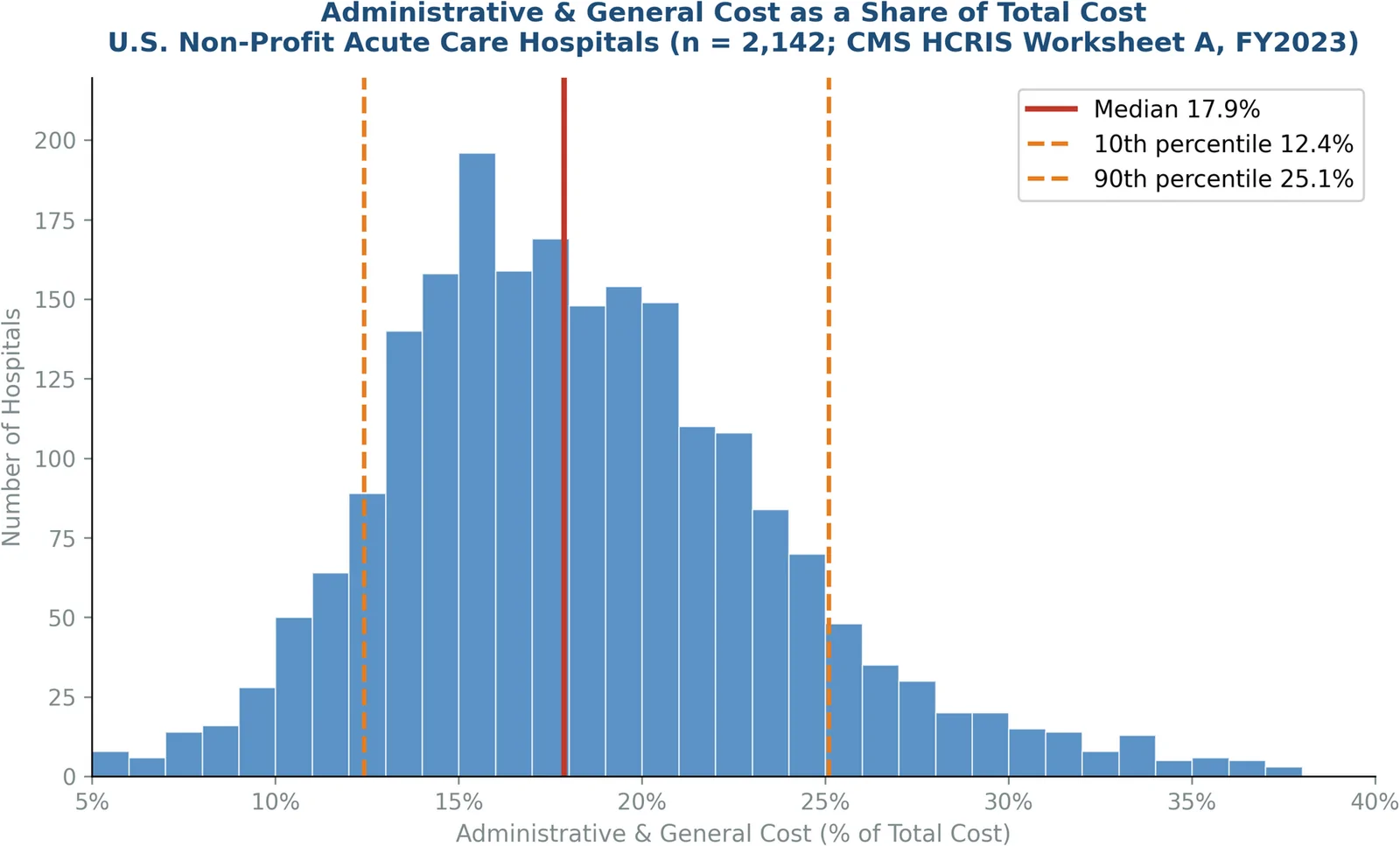
Distribution of Administrative and General Cost as a Share of Total Cost, U.S. Non-Profit Acute Care Hospitals, FY2023 (*n* = 2,142). Source: Author’s analysis of CMS HCRIS Worksheet A (Administrative and General cost center), FY2023. The 2.0× gap between the 90th and 10th percentile hospitals demonstrates that administrative cost intensity is not structurally fixed

Among 2,142 non-profit acute care hospitals in FY2023, median A&G cost was 17.9% of total cost (Fig. 4). This figure sits below the broad 25–49% range reported in the literature, as expected for a narrow cost-center measure, and provides a conservative, replicable baseline. The central finding is the variation: A&G cost ranged from 12.4% at the 10th percentile to 25.1% at the 90th percentile, a 2.0-fold gap between the leanest and least-lean deciles of hospitals sharing the same ownership type, facility type, and regulatory environment. If administrative cost intensity were structurally determined by the nature of acute care, this variation would not exist. Its magnitude is the empirical signature of the accountability vacuum the AAG describes.

### Result 2: Administrative cost share differs by ownership

Non-profit hospitals’ median A&G share (17.9%) was significantly lower than for-profit hospitals (20.3%; Mann-Whitney *p* < 0.001) and higher than government hospitals (15.4%; *p* < 0.001) (Table 1). The for-profit/non-profit difference is consistent with profit-driven overhead structures; the gap above government hospitals is notable given non-profits’ charitable obligations and warrants the kind of activity-level scrutiny the framework proposes. These differences persisted across all three fiscal years.

**Table 1:** Administrative and general cost as a share of total cost by hospital ownership

Measure (non-profit, A&G cost center)	FY2021	FY2022	FY2023
Hospitals analyzed (n)	2091	2121	2142
Median A&G share of total cost	18.2%	17.8%	17.9%
10th–90th percentile range	12.3%–25.1%	12.0%–25.2%	12.4%–25.1%
For-profit median (comparison)	19.7%	19.5%	20.3%
Government median (comparison)	15.6%	15.2%	15.4%

U.S. Short-Term Acute Care Hospitals, FY2021–FY2023. Source: Author’s analysis of CMS HCRIS Worksheet A. Non-profit vs. for-profit and vs. government differences are significant in each year (Mann-Whitney p < 0.001)

### Result 3: The measure is stable across years

Median non-profit A&G share was 18.2% (FY2021), 17.8% (FY2022), and 17.9% (FY2023), and the 10th–90th percentile spread was essentially unchanged (Table 1), indicating the variation finding is structural rather than a single-year artifact. These findings provide direct empirical support for the governance diagnosis. The wide within-ownership variation is precisely what principal-agent theory predicts when agents face heterogeneous and generally weak accountability. In the absence of binding measurement, cost structures drift according to local factors rather than converging toward efficiency. The findings do not by themselves establish the causal governance mechanism, that would require board-level governance data linked to cost outcomes, which we identify as a priority for future research, but they are consistent with the framework’s prediction and inconsistent with the competing dominant cultural claim that administrative cost is structurally fixed. Two limitations bound interpretation in this analysis is: (i) the A&G cost center does not capture administrative activity embedded in clinical departments (biasing our estimate downward relative to broad measures), and (ii) cross-ownership differences may partly reflect unobserved differences in case mix and teaching intensity that risk-adjusted analysis would address.

## A symmetric management accounting framework

The tools required to close the AAG already exist and are routinely applied to clinical departments in the same hospitals where administrative departments escape equivalent scrutiny. Activity-Based Costing (ABC) was formalized by Cooper and Kaplan and has an extensive healthcare evidence base, including a systematic review identifying more than 740 healthcare applications [23, 24]; the Balanced Scorecard by Kaplan and Norton has been widely adapted to healthcare [25]; and Zero-Based Budgeting (ZBB) has corporate and public-sector precedent. What is missing is symmetric application of these methodologies. Table 2 summarizes a five-step framework applied to HR and Workforce Administration, chosen because it has a direct, quantifiable link to the hospital’s largest cost driver, clinical labor, and because it designs the very evaluation systems applied to clinical staff.

**Table 2:** Five-step symmetric management accounting framework

Step	Tool	Application to administration	Key output
1	Activity-Based Costing (ABC)	Trace administrative costs to specific activities using existing HRIS/ERP data	Cost-per-output by activity vs. published benchmarks
2	Downstream cost attribution	Attribute outcomes (e.g., turnover cost) to the administrative function responsible.	Avoidable cost identified and assigned
3	Responsibility center reclassification	Reclassify administrative units from overhead pool to formal cost centers	Monthly variance reporting equivalent to clinical units
4	Balanced Scorecard	KPIs across financial, process, stakeholder, and learning dimensions	Symmetric quarterly reporting to the board
5	Zero-Based Budgeting	Require activity-level justification from first principles	Budget insulation removed; resistance itself diagnostic

HRIS = Human Resource Information System; ERP = Enterprise Resource Planning; KPI = Key Performance Indicator

We illustratively applied this framework to a 350-bed model hospital where the framework attributes avoidable nurse-turnover cost to the HR function responsible instead of current standard of distributing across clinical budgets as agency and overtime expense. Because turnover is multifactorial in nature (labor market, unit culture, compensation, and management factor in), we present this as a sensitivity range rather than a point estimate (Table 3). The illustrative model is explicitly not an empirical claim about any specific institution; it demonstrates the framework’s logic and order of magnitude (Appendix in Supp Doc). The savings projected are gross of implementation cost; net savings will require subtracting the technology, training, and change-management costs discussed below, which we do not model and could be substantial.

**Table 3:** Sensitivity analysis of avoidable annual turnover cost

Scenario	Cost per turnover	Turnover gap	Attribution to HR	Avoidable annual cost (gross)
Low	$40,000	8 pp	50%	$0.56 M
Base	$52,000	8 pp	100%	$2.91 M
High	$65,000	8 pp	100%	$3.64 M

(Illustrative 350-Bed Model). Costs are gross of implementation expense. The 50% low-attribution scenario reflects that turnover has causes beyond HR performance. pp = percentage points

## Technology and implementation: opportunities and barriers

All data rich environments are experiencing an increasing integration of artificial intelligence (AI) to streamline repetitive processes. In this current environment a reasonable question is whether AI is necessary to operationalize this framework? For the core analysis, the answer is no: the ABC attribution, responsibility-center reclassification, and balanced-scorecard reporting in Table 2 can be performed with standard management-accounting and data-analysis methods using data hospitals already hold, and we recommend institutions begin there rather than waiting for advanced technology. However, for operationalization across several administrative departments in a systemwide manner AI can reduce the recurring operational burden that has historically prevented these methods from being applied at scale continuously rather than as one-off exercises. Thus, AI applications can provide prospective efficiency enhancements, a hypothesis that is aggressively being tested in the healthcare management.

Machine-learning models have predicted nurse turnover with reported AUC of 0.69–0.78 in U.S. data and higher accuracy in single-institution studies elsewhere, though these are research-setting results, not operational deployments, and turnover prediction is only one input among many organizational and labor-market drivers [26, 27]. Process mining has peer-reviewed support for workflow optimization in healthcare and could in principle automate activity-cost tracing, though evidence in administrative settings is limited to case studies [28]. Natural-language processing, real-time dashboards, and AI-augmented budgeting are proposed by analogy and remain to be validated.

Realistic implementation must confront substantial barriers, which prior enthusiasm for health-IT solutions has often underestimated. These include limited interoperability between HRIS, ERP, and financial systems; data-quality and completeness problems in administrative records; cybersecurity and privacy risks from integrating sensitive workforce and financial data; workforce resistance, particularly from the administrative functions whose performance would become visible; and regulatory and compliance requirements governing data use. Sociotechnical and organizational-change theory both predict that the social and institutional barriers especially resistance from newly measured units and the leadership incentives that sustain the status quo will frequently be more binding than the technical ones [29, 30]. The framework’s value lies primarily in the governance restructuring where board-level mandate for symmetric reporting will makes measurement consequential. This can be achieved by strategic planning and systemwide implementation.

## Practical guidance

- **For boards**: Request administrative cost as a share of total cost, benchmarked against peers. Establish a board committee, with clinical-leader membership, that receives administrative performance data of equivalent granularity to clinical data, and commission a zero-based review of one administrative department.
- **For clinical leaders**: Document the downstream cost of administrative underperformance in financial terms and present it with attribution; the compliance and documentation burden generated by unaccountable administrative processes is both a cost and a workforce-retention issue [29].
- **For finance leadership**: Begin the ABC analysis pilot for one administrative department using existing data and standard methods in the next budget cycle and build administrative cost ratios into routine board reporting. An interactive calculator for institution-specific illustrative modeling is provided as a supplementary file.

## Limitations and future research

The study is an initial attempt to provide a structure to an increasingly bigger problem in US Healthcare system, if left unaddressed will lead to catastrophic consequences. However, our study has limitations. The A&G cost center understates total administrative cost by excluding billing, insurance-related, and clinically embedded administrative activity; our 17.9% figure is therefore a conservative lower bound, not directly comparable to the broader 25–49% literature estimates. The cross-sectional variation is hypothesis generating but does not prove the principal-agent mechanism. Establishing causal governance effects will require board-level governance data linked to cost outcomes. Cross-ownership differences may partly reflect case mix and teaching intensity not captured here.

The savings model is illustrative only using the publicly available SHRM benchmarks, gross implementation cost, and not validated against any institutional data. Implementation costs (technology, training, change management) that will materially reduce net savings were not modeled. AI application data is quite immature and is still evolving. These are prospective and grounded in adjacent evidence, not validated in administrative accountability settings.

Future research needs to address risk-adjusted analysis of administrative cost using linked clinical and Worksheet A detail; cross-sectional measurement of AAG indicators linked to board governance data; case-study implementation of the framework with pre-post measurement; and cost-effectiveness analysis incorporating implementation costs.

## Conclusion

In summary we found that administrative cost intensity varies twofold among U.S. non-profit acute care hospitals of the same type and sector, a finding inconsistent with the claim that high administrative cost is structurally inevitable and consistent with the accountability vacuum we term the Administrative Accountability Gap. The tools to close it are readily available in management literature; the barrier is most likely governance. Boards must impose on administrators the same accountability they impose on the clinical staff who deliver the care that justifies the institution’s charitable existence. The empirical findings here provide a replicable baseline; however institutional validation is the necessary next step.

## Supplementary Information

Below is the link to the electronic supplementary material.

Supplementary Material 1

## Data Availability

Dey M. Administrative Cost Impact Calculator: An Interactive Supplementary Tool for Non-Profit Acute Care Hospital Management Accounting Research [Software]. GitHub; 2026. Available at: https://mahuadey-tech.github.io/Hospital-admin-calculator/.

## Declarations

### Use of AI tools

The author used a large language model (Claude, Anthropic) for model development, code generation for the HCRIS analysis, figure preparation, and editing. The author designed the study, directed and verified all analyses, confirmed all numerical results against the source data, and takes full responsibility for the content and integrity of the work. No AI system is listed as an author or cited as a source, consistent with ICMJE and COPE guidance on AI use.
