# Supplementary material for: Who measures the measurers: Closing the administrative accountability gap in non-profit acute care hospitals
Source: BMC Health Serv Res. 2026 Jul 9;26:1358. doi: 10.1186/s12913-026-15079-9 (PMC13637122; doi:10.1186/s12913-026-15079-9)
Supplement: Supplementary file 1 [file 12913_2026_15079_MOESM1_ESM.docx]

**Supplementary Material**

**Appendix S1. Detailed CMS HCRIS Data and Methods**

This appendix documents the data extraction and analysis underlying the empirical results reported in the main text, in sufficient detail for independent replication from the publicly available CMS Healthcare Cost Report Information System (HCRIS) Hospital 2010 Cost Report files.

**S1.1 Data source and files**

We obtained the complete HCRIS Hospital 2010 Cost Report public-use files for federal fiscal years 2021, 2022, and 2023 from the Centers for Medicare & Medicaid Services. For each year we used three linked files:

- **Report file (RPT):** one record per cost report, providing the report record number, the provider CCN, and the Type-of-Control code (Worksheet S-2, line 21).
- **Numeric file (NMRC):** the reported dollar values, keyed by report record number, worksheet code, line number, and column number.
- **Alphanumeric file (ALPHA):** the worksheet line/column labels, used to confirm that Worksheet A, line 00500 corresponds to “Administrative & General.”

**S1.2 Cohort definition**

We restricted the analytic sample to short-term acute care hospitals (CCN facility-type code STH). Ownership was classified from the Type-of-Control code:

| **Type-of-Control codes** | **Classification** |
| --- | --- |
| 1–2 (Voluntary Nonprofit — Church / Other) | Nonprofit |
| 3–6 (Proprietary) | For-profit |
| 7–13 (Governmental) | Government |

*Table S1. Ownership classification from the HCRIS Type-of-Control field.*

**S1.3 Administrative cost measure**

Administrative cost was measured as the Administrative and General (A&G) cost center from Worksheet A, expressed as a share of total facility cost. Specifically, from the numeric file we extracted, per report:

A&G net cost	= Worksheet A000000, line 00500, column 00700

Total net cost = Worksheet A000000, line 20000, column 00700

A&G share	= (A&G net cost) / (Total net cost)

Column 00700 is the net cost column of Worksheet A (salaries + other costs, net of reclassifications and adjustments). We mapped each report to its ownership class via the report record number in the RPT file.

**S1.4 Exclusions and final sample**

We excluded reports with non-positive total cost and restricted the A&G share to the economically valid 5–40% range to remove cost-allocation artifacts (affecting under 1% of non-profit observations — 17 of 2,159 in FY2023). The final analytic sample comprised:

| **Fiscal year** | **Non-profit STH hospitals analyzed** |
| --- | --- |
| FY2021 | 2,091 |
| FY2022 | 2,121 |
| FY2023 | 2,142 |
| Total | 6,354 |

*Table S2. Final analytic sample of non-profit acute care hospitals by year.*

**S1.5 Statistical analysis**

Distributions were summarized with medians and 10th/90th percentiles given right-skew. Between-ownership differences were tested with the Mann-Whitney U test. Analyses were performed in Python (pandas, scipy). Analysis code is available from the author on reasonable request.

**S1.6 Limitations of the measure**

- **Scope:** The A&G cost center excludes billing, insurance-related, and clinically embedded administrative activity; our estimate is a conservative lower bound relative to the broad 25–49% literature definitions.
- **Risk adjustment:** Cross-ownership differences are unadjusted for case mix, teaching status, and service scope; risk-adjusted analysis using linked clinical data is a stated future direction.
- **Causality:** The cross-sectional design supports the framework’s prediction but does not establish causal governance effects, which would require board-level governance data linked to cost outcomes.

**Appendix S2. Interactive Administrative Cost Calculator**

An interactive calculator accompanies this manuscript as a teaching and institution-specific modeling tool. It is available at: **https://mahuadey-tech.github.io/Hospital-admin-calculator/**.

The calculator lets users enter hospital size, administrative staffing share, and turnover rate, and returns illustrative cost and savings estimates. Importantly, its benchmark panel offers a **measure toggle** that distinguishes the narrow Administrative & General cost center (from our CMS HCRIS analysis; non-profit median 18%) from the broad administrative-cost definition reported in the literature (25–49%). This mirrors the dual framing used in the main text and prevents the two measures from being conflated.

**Figure S1.** Interactive administrative cost calculator (overview tab). *All calculator outputs are illustrative; institution-specific values vary.*

**S2.1 Calculator model assumptions**

The calculator’s illustrative model uses the following default parameters, drawn from published industry benchmarks. These are illustrative and adjustable by the user:

| **Parameter** | **Default value** | **Source basis** |
| --- | --- | --- |
| Budget per bed | $1.14M | CMS HCRIS operating-cost distributions |
| FTE per bed | 6.3 | AHA Annual Survey; HCRIS |
| Avg. administrative salary (loaded) | $72,000 × 1.38 overhead | SHRM benchmarks |
| Cost per RN turnover incident | $52,000 | NSI Nursing Solutions (2023) |
| Best-practice turnover target | 14% | NSI Nursing Solutions (2023) |

*Table S3. Default model parameters in the interactive calculator. All figures illustrative.*

**Appendix S3. Turnover Cost Sensitivity Analysis (Full)**

The avoidable-turnover sensitivity analysis summarized in main-text Table 2 is reproduced here with full assumptions. All figures are gross of implementation cost.

| **Scenario** | **Cost / incident** | **Turnover gap** | **Attribution to HR** | **Avoidable annual cost (gross)** |
| --- | --- | --- | --- | --- |
| Low | $40,000 | 8 pp (22%→14%) | 50% | $0.56M |
| Base | $52,000 | 8 pp (22%→14%) | 100% | $2.91M |
| High | $65,000 | 8 pp (22%→14%) | 100% | $3.64M |

*Table S4. Avoidable annual nurse-turnover cost under low/base/high assumptions for an illustrative 350-bed hospital. The 50% low-attribution scenario reflects that turnover is multiply determined (labor market, unit culture, compensation, management) and not attributable to HR performance alone.*
